# Pessaries

**Published:** 1864-09

**Authors:** M. M. Eaton

**Affiliations:** Peoria


### PESSARIES.

By M. M. EATON, M. D., of Peoria.

Doubtless no physician, of any considerable experience in the treatment of females, is entirely satisfied with any pessary before the Medical public. Most of those now in use have their friends and advocates, though no one of them is all that is desired; and I think that every philanthropic, well educated physician has been annoyed because no better relief can be afforded to one of the most distressing, though common, complaints to which the female is liable—one so common that we are almost daily consulted about it—one giving rise, not only to suffering, but barrenness, which often so much disturbs conjugal felicity, viz., Prolapsus Uteri.

In order to treat successfully this complaint we must be conversant with its cause, the removal of which should be our first study. In Prolapsus Uteri, the strength of the vaginal walls, the broad and round ligaments, is disproportionate to the weight of the Uterus. This weakness is the result of irritation from various causes in most instances, though occasionally from want of, or entire absence of, irritation. Getting up too soon after confinement, while the vagina is relaxed and the womb heavy, is a frequent cause.

Now, in view of the above facts, it is clearly our duty to reinstate the Uterus *in situ*, retain it there, and strengthen the vaginal walls, while at the same time we support the abdominal viscera with suitable bandages and pads, so as to relieve the womb as much as possible of all superincumbent weight.

On the operation of replacing the organ I will not dwell, as it is generally an easy operation, soon effected by continuous, though gentle pressure on the os uteri with two fingers, having the patient in a recumbent posture, with the hips elevated.

When this is accomplished the patient should not rise for several days, during which time weak astringent washes should be used in the vagina, several times a day, with a rubber syringe, having the patient over a bed-pan the while.

This treatment would effect a cure of itself if long enough maintained, but as patients are restless and impatient of restraint, they will hardly submit to this confinement more than a few days, and as the vagina has not become strong enough to bear the weight of the womb, prolapsus is again the result. To remedy this, various contrivances have been invented to place in the vagina to support the Uterus, all of which, that I have seen or heard of, are to be objected to seriously, in that they simply alleviate but do not cure. Some distend the vagina making it weaker rather than stronger; others irritate the os uteri; others are cumbersome, and have to be removed and readjusted daily. Now, in view of all this, I can agree with Dr. Rigby and others, that these various pessaries have done more mischief than good. But a plan occurred to me some years ago that an instrument could be invented that would answer all the indications, and still liable to none of the objections I have enumerated. I accordingly submit to the Medical profession the following described instrument, hoping it may be found convenient to the physician and a comfort and means of cure to many afflicted daughters of Eve.

The instrument proper is pear-shaped, made of any convenient material, about one and a half inches in diameter, with a cavity, about one inch wide, passing through, and terminating below in an opening, half an inch wide. Around this orifice are inserted three standards which meet externally, and are fastened into a cross piece, placed transversely from before backwards, which divides into two pieces, before and behind, so as to surround the meatus urinarius and the anus, these uniting to form a handle or loop, to which is attached elastic straps passing upward and fastened to a waist band buckled above the hips.

This instrument can be worn indefinitely without removal, as it does not interfere with defecation or micturition, or with the use of the small pipe of a female syringe, which we need, for several weeks, to continue the cool astringent washes. It does not distend the vagina, it holds the os uteri steady, and will not irritate it. Through the opening in the instrument in which the *Os* rests, the menstrua may be discharged. Hence it seems to me that by the use of this instrument and proper collateral treatment, this distressing malady may be speedily cured, so as to need no treatment whatever. More than this, I believe that this instrument will, after a while, effect a cure of itself unaided, as it keeps the womb *in situ*, and gives time for the vagina to become strong. The old stem-globe pessary only holds the womb up by pressing up between the os uteri and the walls of the vagina. The womb would not rest on it as it has been constructed, and when a dent has been made on the superior surface, it would not hold the womb directly, but simply holds the secretions, and necessitates its frequent removal. It also interferes, much more than my instrument, with defecation and urination; in fact, my instrument interferes seriously with nothing but copulation, which, by the way, it is well to prohibit.

